# Regulating Oxygen Ion Transport at the Nanoscale to Enable Highly Cyclable Magneto-Ionic Control of Magnetism

**DOI:** 10.1021/acsnano.3c01105

**Published:** 2023-03-27

**Authors:** Zhengwei Tan, Zheng Ma, Laura Fuentes, Maciej Oskar Liedke, Maik Butterling, Ahmed G. Attallah, Eric Hirschmann, Andreas Wagner, Llibertat Abad, Nieves Casañ-Pastor, Aitor F. Lopeandia, Enric Menéndez, Jordi Sort

**Affiliations:** † Departament de Física, Universitat Autònoma de Barcelona, 08193 Cerdanyola del Vallès, Spain; ‡ Institut de Ciència de Materials de Barcelona, CSIC, Campus UAB, 08193 Bellaterra, Barcelona, Spain; # Centre Nacional de Microelectrònica, Institut de Microelectrònica de Barcelona-CSIC, Campus UAB, 08193 Bellaterra, Barcelona, Spain; ¥ Institute of Radiation Physics, Helmholtz-Zentrum Dresden - Rossendorf, Dresden 01328, Germany; △ Institució Catalana de Recerca i Estudis Avançats (ICREA), Pg. Lluís Companys 23, E-08010 Barcelona, Spain; ¶ Catalan Institute of Nanoscience and Nanotechnology (ICN2), CSIC and BIST, Campus UAB, Cerdanyola del Vallès, 08193 Barcelona, Spain

**Keywords:** magneto-electricity, voltage control of magnetism, magneto-ionics, transition metal oxide, ion diffusion

## Abstract

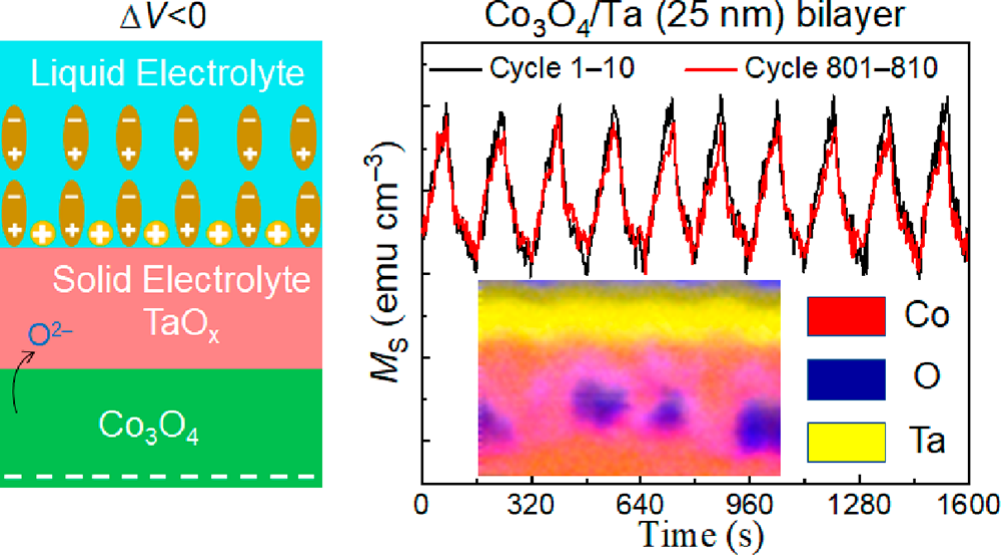

Magneto-ionics refers to the control of magnetic properties of materials through voltage-driven ion motion. To generate effective electric fields, either solid or liquid electrolytes are utilized, which also serve as ion reservoirs. Thin solid electrolytes have difficulties in (i) withstanding high electric fields without electric pinholes and (ii) maintaining stable ion transport during long-term actuation. In turn, the use of liquid electrolytes can result in poor cyclability, thus limiting their applicability. Here we propose a nanoscale-engineered magneto-ionic architecture (comprising a thin solid electrolyte in contact with a liquid electrolyte) that drastically enhances cyclability while preserving sufficiently high electric fields to trigger ion motion. Specifically, we show that the insertion of a highly nanostructured (amorphous-like) Ta layer (with suitable thickness and electric resistivity) between a magneto-ionic target material (*i*.*e*., Co_3_O_4_) and the liquid electrolyte increases magneto-ionic cyclability from <30 cycles (when no Ta is inserted) to more than 800 cycles. Transmission electron microscopy together with variable energy positron annihilation spectroscopy reveals the crucial role of the generated TaO_*x*_ interlayer as a solid electrolyte (*i.e*., ionic conductor) that improves magneto-ionic endurance by proper tuning of the types of voltage-driven structural defects. The Ta layer is very effective in trapping oxygen and hindering O^2–^ ions from moving into the liquid electrolyte, thus keeping O^2–^ motion mainly restricted between Co_3_O_4_ and Ta when voltage of alternating polarity is applied. We demonstrate that this approach provides a suitable strategy to boost magneto-ionics by combining the benefits of solid and liquid electrolytes in a synergetic manner.

With the advent of global phenomena such as the Internet of Things, artificial intelligence, machine learning, or Big Data, the demand for highly functional and energy-efficient miniaturized microelectronic components is growing exponentially.^1,2^ Spintronic systems^3,4^ operated using electric currents through spin-transfer torque^5,6^ or spin–orbit torque^7,8^ effects are becoming key elements for next-generation nanoelectronics with enhanced memory and information processing capabilities. However, magnetization switching using electric current involves an undesirable Joule heating effect, which is detrimental to energy efficiency.^9,10^ An interesting alternative is to modulate magnetic properties by applying electric fields instead of electric current, thus minimizing power dissipation. This has rapidly evolved into a whole area of research referred to as voltage control of magnetism (VCM).

Magneto-ionics refers to a particular mechanism for VCM in which voltage-driven ion transport (of, *e.g.*, O^2–^,^11−15^ Li^+^,^16^ F^–^,^17^ H^+^,^18−21^ or N^3–^^22−26^ species) leads to a large and controllable modulation of magnetism without the need of strain transfer. This is different from voltage-controlled strain-mediated multiferroic heterostructures, which are less convenient for spintronics because repeated voltage actuation can lead to mechanical fatigue and eventual device failure. Archetypical magneto-ionic structures comprise a gate electrolyte (either solid or liquid)^27,28^ in contact with a ferromagnetic (FM) target material. Voltage is applied across the electrolyte, using the FM film as a working electrode and causing a voltage polarity-dependent insertion/removal of ions into/from the target material. In this way, magnetic properties, such as saturation magnetization,^11,22^ magnetic anisotropy and coercivity,^12,18^ exchange bias field,^13,29^ or skyrmion generation/suppression,^20^ among others, can be reversibly controlled, making magneto-ionic materials highly promising for ultra-low-power magnetic devices. An extreme case is when voltage induces a complete reversible transition between FM and nonmagnetic states, leading to voltage-driven ON–OFF switching of ferromagnetism. This has been reported in a number of magneto-ionic systems, such as Co_3_O_4_,^11,14,30^ Li^+^-intercalated α-Fe_2_O_3_,^31^ F^–^-intercalated La_2–2*x*_Sr_1+2*x*_Mn_2_O_7_,^17^ CoN,^26,28^ CoMnN,^25^ or α-Co(OH)_2_.^32^ ON–OFF switching of the ferromagnetic state has been also induced by electrostatic surface charging in FeS_2_.^33^

In most magneto-ionic systems, the source of the moving ions is electrolytes (*e.g*., GdO_*x*_, HfO_*x*_, propylene carbonate with dissolved LiPF_6_ or KI) that are in direct contact with pristine FM or ferrimagnetic layers (*e.g.*, Co, Fe, Fe_2_O_3_), whose properties are manipulated with voltage.^27,34−36^ Magneto-ionic systems based on O^2–^ insertion from an external electrolyte to a target FM material often suffer from slow dynamics at room temperature and irreversible compositional/structural changes in the FM phase, eventually leading to degradation and limited cyclability.^12^ In addition, for relatively thick films, it is challenging to achieve a fully OFF magnetic state with voltage due to the limited penetration of the external ions toward the interior of the FM target layers.^37^ Recently, smaller ions (*e.g.*, H^+^, F^–^, or Li^+^) have been introduced to achieve faster and more cyclable voltage-induced manipulation of magnetic properties. Fast, reversible, and cyclable tuning of perpendicular magnetic anisotropy,^18^ Dzyaloshinskii–Moriya interaction,^38,39^ or ferrimagnetic spin textures^40^ has been achieved through chemisorbed O^2–^ or H^+^ ion species. However, systems relying on H^+^ are either sensitive to environmental conditions (*e.g*., humidity) or restricted by their incompatibility with traditional complementary metal-oxide semiconductor (CMOS)-based devices (*i.e*., standard fabrication processes for semiconductor devices, such as metal oxide field-effect transistors).^28^ An alternative approach is to use structural oxygen or nitrogen, self-contained in the target materials, as the source of the moving ions. Examples of such materials are Co_3_O_4_,^11^ CoN,^22,26^ or CoMnN^25^ films. These target materials, which are CMOS compatible, exhibit an initially fully OFF (*i*.*e*., paramagnetic) state and provide “ready-prepared” lattice sites for ion diffusion, allowing for net magneto-ionic generation of ferromagnetism by voltage-triggered O^2–^ or N^3–^ ion motion from the films toward a neighboring electrolyte. Unfortunately, achieving a high magneto-ionic cyclability in these materials (*i.e*., removing and reinserting the O^2–^/N^3–^ ions many times by switching voltage polarity) remains a challenge.

To induce magneto-ionics, either solid or liquid electrolytes can be utilized. Solid electrolytes (with ultrathin dielectric layers) are preferred for solid-state spintronics. Ultrathin solid dielectric layers are needed to induce sufficiently large electric fields under moderate applied voltages. However, at such small thicknesses, difficulties arise to withstand high electric fields without electric pinholes. Moreover, thin solid electrolytes offer a limited ion buffering capability (*i.e*., they easily become saturated with ions and cannot sustain stable ion transport during long-term operation, especially when the magneto-ionic layer is thicker than the solid electrolyte layer). Liquid electrolytes are convenient for other magnetoelectric applications, such as magnetophoresis/microfluidics^41^ or to emulate neuromorphic functionalities (the brain operates in a liquid environment).^15,26^ Owing to the formation of the “electric double layer”, whose thickness is <1 nm, liquid electrolytes are able to generate ultralarge electric fields (hundreds of MV cm^–1^) at the interface between the liquid and the target magneto-ionic layer, without electric pinholes.^27^ Liquid electrolytes may also be good ion reservoirs.^11,14,22^ However, when voltage is applied, the mobile ions released from the target layer into the liquid can travel long distances toward the counter electrode, making their reintroduction into the magneto-ionic layer (with voltage of opposite polarity) difficult. In addition, at the counter electrode, if sufficiently high voltage is applied, the dissolved ions (*e.g*., O^2–^, H^+^, or N^3–^) can transform into the corresponding oxygen, hydrogen, or nitrogen gases and be released to the atmosphere in the form of bubbles. These effects are difficult to control and are highly detrimental for magneto-ionic reversibility and endurance. Restricting ion transport within the magneto-ionic layers could avoid these problems and is expected to improve the magneto-ionic cyclability.

Here, we propose an improved nanoscale-engineered magneto-ionic structure that results from inserting an amorphous Ta layer (which gets spontaneously passivated in air) between the Co_3_O_4_ film (magneto-ionic target material) and propylene carbonate, PC (liquid electrolyte). Upon application of negative voltage, O^2–^ ions migrate from Co_3_O_4_ to Ta, promoting the formation of TaO_*x*_ (Ta is a good oxygen getter), which acts as a thin solid electrolyte with good ionic conductivity. This architecture preserves sufficiently high electric fields to trigger ion motion while allowing to repeatedly induce ON–OFF switching of ferromagnetism at room temperature. By optimizing the thickness of the Ta layer, a drastic increase of cyclability is achieved in Co_3_O_4_/Ta (25 nm)/PC (>800 cycles) compared to Co_3_O_4_/PC with no Ta insertion (<30 cycles). The Ta layer hinders oxygen ions from entering the liquid electrolyte and allows for O^2–^ redistribution inside the Co_3_O_4_ and Ta layers, as assessed by high-angle annular dark-field scanning transmission electron microscopy (HAADF-STEM) and electron energy loss spectroscopy (EELS). Positron annihilation spectroscopy is used to precisely characterize the defect structure in Ta, which strongly contributes to the O^2–^ ion transport. An increase of the defect size during biasing is observed. The reduced electric conductivity of the passivated amorphous Ta layer is important to allow the penetration of the electric field inside the Co_3_O_4_ layer and the concomitant O^2–^ ion transport. Since O^2–^ diffusion becomes mainly restricted within the Co_3_O_4_/TaO_*x*_ structure (instead of O^2–^ being released to PC), this greatly improves the efficiency of ion transport in the material, resulting in largely enhanced magneto-ionic cyclability.

## Results and Discussion

**Figure 1:**
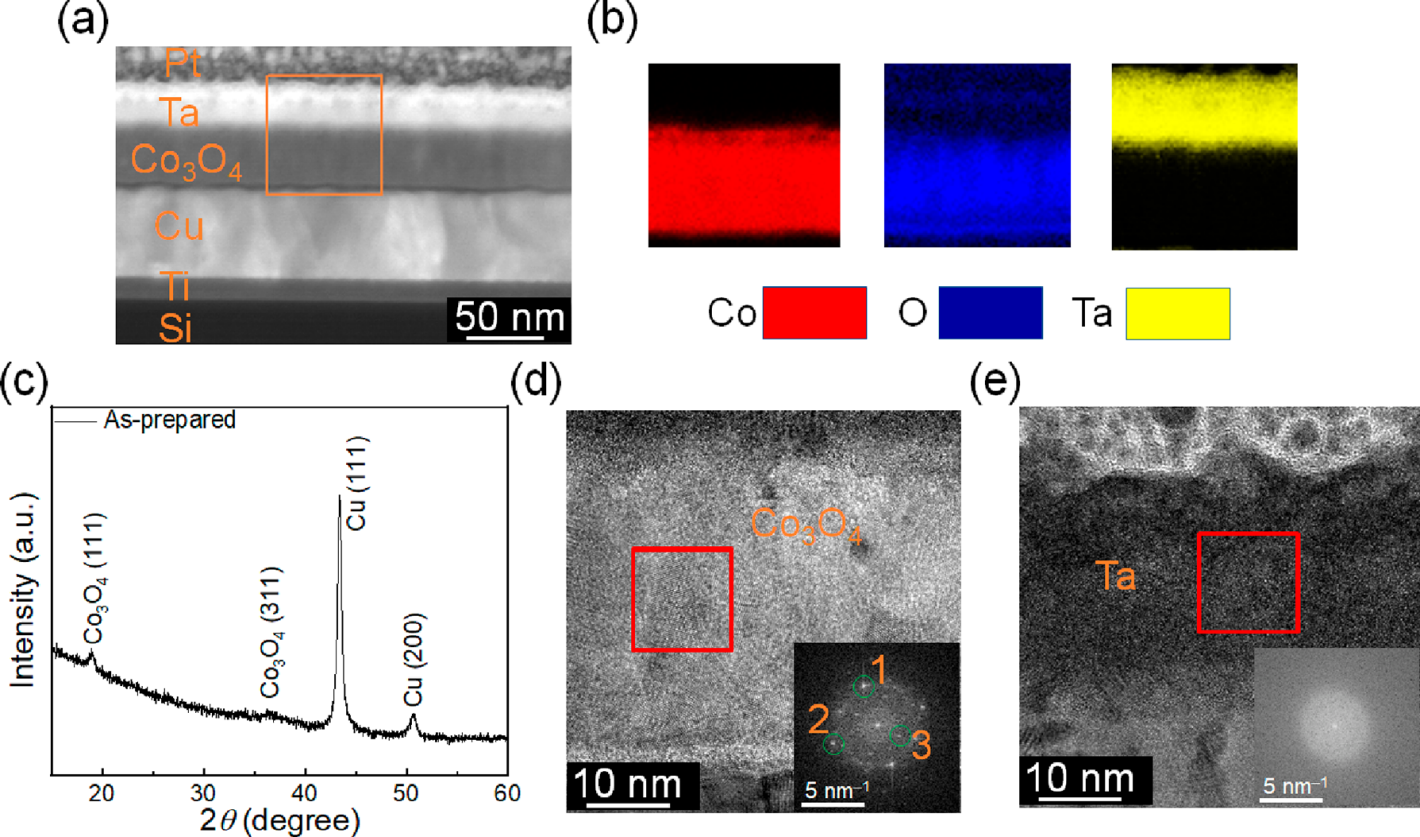
Structural and compositional characterization of as-deposited films. (a) HAADF-STEM micrograph of an as-grown sample. (b) EELS Co (in red), O (in blue), and Ta (in yellow) elemental mappings of the area marked with an orange rectangle in (a). (c) θ/2θ XRD patterns of the as-prepared samples. (d, e) High-resolution TEM images of the cross section of the as-deposited Co_3_O_4_ and Ta films, respectively. The inset shows the fast Fourier transform of the areas marked with red squares. In panel (e), the “1” spot corresponds to an interplanar distance of 0.248 nm and is consistent with (311) Co_3_O_4_ (PDF 00-009-0418) or (111) CoO (PDF 00-001-1227) interplanar distances (0.244 and 0.245 nm, respectively), while spot “2” corresponds to an interplanar distance of 0.225 nm and is unambiguously ascribed to the (200) CoO (PDF 00-001-1227) interplanar distance (0.212 nm). The “3” spot corresponds to an interplanar distance of 0.448 nm, which is consistent with (311) Co_3_O_4_ (0.467 nm, PDF 00-009-0418).

The basic building block of the investigated magneto-ionic system is a 40 nm thick Co_3_O_4_ film grown by DC reactive sputtering at room temperature onto Cu (60 nm)/Ti (20 nm)/[100]Si (725 μm) substrates. The Co_3_O_4_ films were coated with sputtered amorphous Ta protective layers of variable thickness (from 5 to 50 nm), which were left to passivate in air (see Experimental Section). Uncoated Co_3_O_4_ films were also grown as a reference. Figure 1(a) shows a low-magnification HAADF-STEM image of the as-prepared sample with a 25-nm-thick Ta protective capping layer. Clear interfaces between the various layers can be seen, and their thicknesses are in good agreement with the nominal ones. Note that the topmost Pt capping layer was grown only during the TEM lamella preparation (*i.e*., it was not present during magneto-ionic experiments). To investigate the Co, O, and Ta distribution, Co, O, and Ta EELS mappings from the area marked with an orange rectangle in Figure 1(a) were acquired. As shown in Figure 1(b), Co and O elements are homogeneously distributed within the Co_3_O_4_ films. Additionally, some O signal is also detected inside the Ta layer, particularly on its upper part. This is ascribed to surface self-passivation of Ta in air, which is known to result in the formation of a stable TaO_*x*_ top layer.^42,43^Figure 1(c) shows the θ/2θ X-ray diffraction (XRD) pattern of an as-grown film. The XRD peak observed at 2θ ≈ 19.1° is consistent with (111) Co_3_O_4_ (PDF 00-009-0418), whereas that at 2θ ≈ 36.7° could be ascribed to either (111) Co_3_O_4_ or (111) CoO (PDF 00-001-1227). Peaks corresponding to the Cu buffer layer are also detected. Conversely, no traces of Ta are evidenced, suggesting that Ta is highly nanostructured or even amorphous-like. Growth of amorphous Ta by physical deposition methods has been previously reported,^44,45^ and it is promoted by the existence of ions and particles with high kinetic energy when sputtering at sufficiently high gun powers. Amorphous Ta exhibits higher electric resistivity than its crystalline counterparts.^44^ This is beneficial to keep sufficient electric field strength inside the actuated films while voltage is applied, thereby enabling magneto-ionics.

To further investigate the structure of the samples, high-resolution transmission electron microscopy (HRTEM) images of the cross sections of as-prepared Co_3_O_4_ and Ta layers were taken, as shown in Figure 1(d) and (e), respectively. The areas marked with red squares were chosen for fast Fourier transform (FFT) analyses, as shown in the insets. In Figure 1(d), the “1” spot corresponds to an interplanar distance of 0.248 nm and is consistent with (311) Co_3_O_4_ (PDF 00-009-0418) or (111) CoO (PDF 00-001-1227) interplanar distances (0.244 and 0.245 nm, respectively), while spot “2” corresponds to an interplanar distance of 0.225 nm and is unambiguously ascribed to the (200) CoO (PDF 00-001-1227) interplanar distance (0.212 nm). The “3” spot corresponds to an interplanar distance of 0.448 nm, which is consistent with (311) Co_3_O_4_ (0.467 nm, PDF 00-009-0418). This indicates that a mixture of CoO and Co_3_O_4_ phases is plausible in the Co oxide film, in agreement with previously reported results.^15^ For simplicity, in spite of the presence of CoO in particular close to the interface, the Co oxide film is labeled Co_3_O_4_ throughout the article. Remarkably, no spots are observed for Ta, in agreement with its amorphous nature, as also evidenced by XRD (Figure 1(c)).

**Figure 2:**
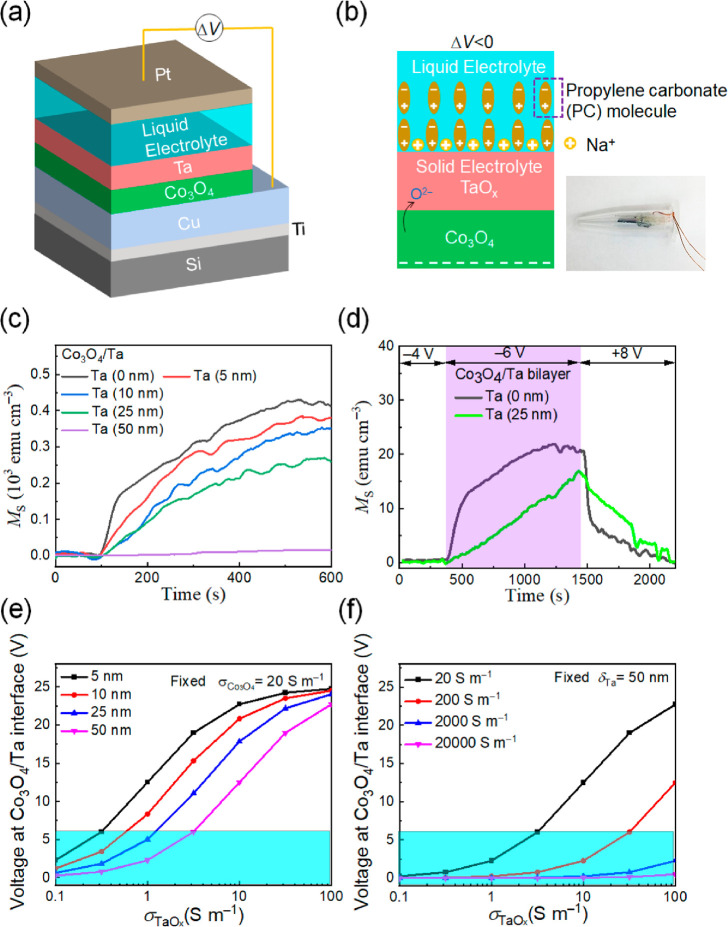
Magneto-ionic characterization of Co_3_O_4_ thin films and Co_3_O_4_/Ta bilayer films under electrolyte gating. (a) Schematic of the designed structure for electrolyte actuation. (b) Left: Sketch of the formation of electric double layer during voltage actuation; right: photograph of the homemade electrolytic cell used to apply voltage to our system, which consists of an Eppendorf filled with propylene carbonate, our sample as working electrode, and a Pt wire counter electrode. (c) Time evolution of the saturation magnetization, *M*_S_, for Co_3_O_4_ thin films and Co_3_O_4_/Ta bilayer films (with a Ta thickness of 5, 10, 25, and 50 nm) under an applied voltage of −25 V. (d) Estimation of the onset voltage required to trigger magneto-ionics in Co_3_O_4_ thin films and Co_3_O_4_/Ta (25 nm) bilayer films, as well as the recovery process. (e) Calculated absolute value of the voltage at the Co_3_O_4_/Ta interface, |Δ*V*_int_|, as a function of the TaO_*x*_ conductivity, σ_TaOx_, when externally applying |Δ*V*| = 25 V for different thicknesses of the Ta layer, δ_Ta_, and a fixed Co_3_O_4_ conductivity, σ_Co3O4_ = 20 S m^–1^. (f) Calculated interface voltage as a function of σ_TaOx_, for different values of conductivity of σ_Co3O4_ and a fixed Ta thickness (δ_Ta_ = 50 nm). The regions highlighted with a cyan rectangle in (e) and (f) are those where |Δ*V*_int_| would be below the magneto-ionic threshold voltage of the system.

**Figure 3:**
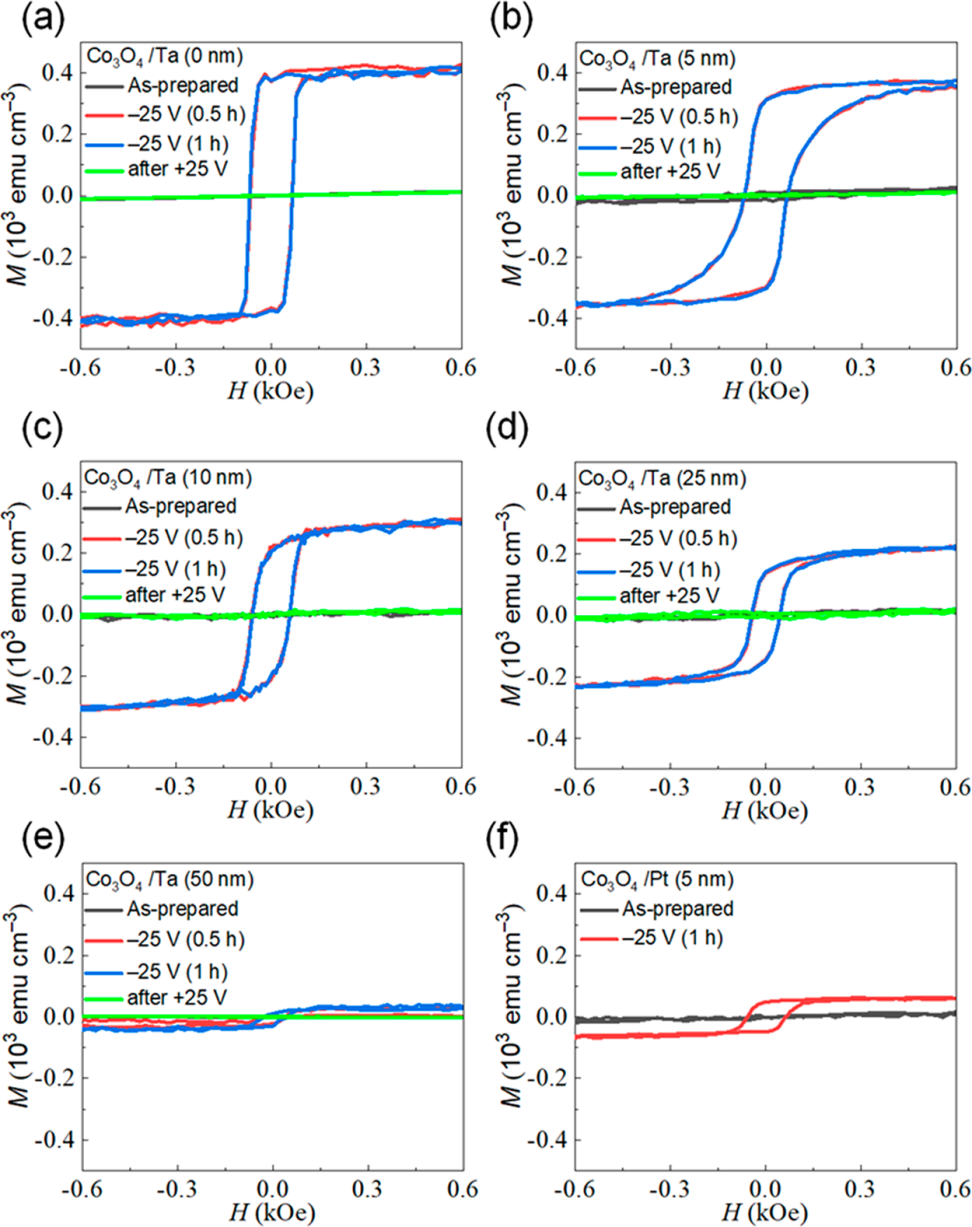
In-plane VSM hysteresis loops (each lasting 30 min) of the as-prepared Co_3_O_4_/Ta films (black), the films biased under −25 V (for 30 min in red and for 1 h in blue), and subsequently recovered after applying +25 V for 30 min. The different panels correspond to different Ta thicknesses: (a) 0 nm, (b) 5 nm, (c) 10 nm, (d) 25 nm, (e) 50 nm. Panel (f) corresponds to Co_3_O_4_/Pt without a Ta interlayer (with a Pt thickness of 5 nm).

To induce magneto-ionics, electrolyte gating was performed in a capacitor-like configuration (Figure 2(a))^14^ using a platinum wire as counter electrode and an aprotic, anhydrous polar liquid electrolyte composed of propylene carbonate with Na^+^ and OH^–^ solvated species.^27,46,47^ When voltage is applied, a sub-nm-thick electric double layer forms at the electrolyte side of the electrolyte/Ta interface, allowing for the generation of a high electric field.^47^ This electric field is ultimately responsible for driving oxygen ions from the Co_3_O_4_ to the Ta/TaO_*x*_ layers (as illustrated in Figure 2(b)). Voltage treatments were performed *in situ*, while hysteresis loops were recorded at room temperature by vibrating sample magnetometry (VSM), with an in-plane applied magnetic field. The total measured magnetic moment of the samples (in emu) was normalized to the area of the sample and the nominal thickness of Co_3_O_4_ (to obtain emu cm^–3^). As seen in Figure 3, irrespective of the thickness of the Ta layer (*i*.*e*., 0, 5, 10, 25, or 50 nm), all films in the as-grown state show very little ferromagnetic response (<33 emu cm^–3^, which is equivalent to ≈2.3% the magnetization of pure FCC-Co^48^). This small ferromagnetic signal is common in sputtered Co_3_O_4_^15^ and is likely due to either a small fraction of residual Co clusters that do not become fully oxidized during the sputtering process or substrate contamination. The deposition of Ta onto Co_3_O_4_ has a negligible effect on the initial ferromagnetic signal of the Co_3_O_4_ films. Upon negative biasing at −25 V for 30 min or 1 h, clear hysteresis loops are observed. The generated saturation magnetization (*M*_S_) is maximum for the sample without Ta (around 400 emu cm^–3^, suggesting that 27% of the volume of Co_3_O_4_ gets reduced to Co), and it slightly decreases when a thin Ta interlayer (5–10 nm) is inserted between the Co_3_O_4_ film and the liquid electrolyte. *M*_S_ further decreases for sufficiently thick Ta. Remarkably, magneto-ionic effects when Pt (instead of Ta) is grown onto Co_3_O_4_ are strongly reduced: a small hysteresis loop is observed only for 5 nm Pt (compare Figure 3(f) with (b)), and no ferromagnetic response is obtained for thicker Pt. In all cases, the initial virtually nonmagnetic state can be recovered by applying +25 V for 30 min.

Figure 3 shows that, for all samples, most of the ferromagnetic response is induced in less than 30 min, since *M*_S_ does not further increase when applying voltage for 1 h. The time evolution of *M*_S_ for all samples under −25 V is shown in Figure 2(c). An external magnetic field of 10 kOe (*i.e*., above the anisotropy field of the generated ferromagnetic counterpart) was applied during these measurements to ensure magnetic saturation. In all films, an immediate increase of *M*_S_ is observed in response to the applied Δ*V*, evidencing a quick onset of the oxygen ionic motion, which leaves metallic ferromagnetic Co behind.^14,15^ The obtained steady-state value of *M*_S_ decreases with the Ta thickness, from 401 emu cm^–3^ to 19 emu cm^–3^ (in agreement with the magnetic hysteresis loops shown in Figure 3(a)–(e)). One important parameter of large significance for device applications is the minimum threshold voltage required to trigger magneto-ionic effects.^22,25^ The onset voltage for Co_3_O_4_ films without and with a 25-nm-thick Ta capping layer was evaluated by subjecting the films to increasing negative voltage steps of −2 V, until *M*_S_ started to increase, as shown in Figure 2(d). The results reveal that the onset voltage is approximately −6 V for both samples, whereas a voltage of +8 V leads to complete recovery in both cases, which agrees with previous works on similar systems.^11^

Figure 2(c) and (d) also show that the rate at which *M*_S_ increases under voltage application is larger for a smaller Ta layer thickness. This, together with the reduction of the steady-state *M*_S_ for thicker Ta, indicates that the effective electric field acting on the Co_3_O_4_ layer becomes lower for larger Ta thickness. This suggests that, for thicker Ta, there is a more severe dissipation of space charge density, while O^2–^ is migrating from Co_3_O_4_ to form TaO_*x*_.^49^

In a first approximation, this effect can be modeled considering the system as a simple voltage divider. The voltage drop at the Co_3_O_4_/Ta interface can be estimated assuming that, during the magneto-ionic process, the Co_3_O_4_ and the newly formed TaO_*x*_ layers act as two resistances connected in series. In this case, the voltage at the Co_3_O_4_/TaO_*x*_ interface is . Considering that *R* = *ρδ*/*A* (where ρ is the resistivity of each material, δ is the film thickness, and *A* is the lateral area of the films), it is straightforward to estimate Δ*V*_int_ as a function of the layers’ thicknesses and the electric conductivities of Co_3_O_4_ and TaO_*x*_. The electrical conductivity of Co_3_O_4_, measured by the Van der Pauw method, is approximately σ_Co3O4_ = 20 S m^–1^.^14^ The conductivity of TaO_*x*_ strongly depends on the oxygen content, and it varies by several orders of magnitude, from 10^5^ S m^–1^ in amorphous metallic Ta^44^ to 10^–6^ S m^–1^ for highly oxidized Ta.^50^ The presence of oxygen in the naturally passivated Ta (Figure 1(b)) can easily bring ρ to values in the range 10^–1^–10^2^ S m^–1^ before any voltage is applied.^51^Figure 2(e) shows the evolution of the calculated interface voltage as a function of σ_TaOx_ for the different Ta film thicknesses. For highly conductive Ta (or TaO_*x*_) spacer layers, Δ*V*_int_ becomes independent of the Ta thickness, which means that any eventual magneto-ionic effect would be independent of the Ta thickness (and Δ*V*_int_ would be always equal to −25 V). However, in this case, the electric field would be highly screened at the interface and would not penetrate inside Ta (since, in metals, the electric field is confined within the Thomas–Fermi screening length, which is typically <0.5 nm).^27^ Thus, for highly metallic interlayers (*e.g*., noble metals like Pt, as in Figure 3(f)), little magneto-ionic effects are expected since there is no driving force (no electric field) to induce ion motion inside the metal.^24^ This is opposite to what happens in semiconductors (*e.g*., TaO_*x*_), where the electric field will penetrate deeper into the layer. Interestingly, for low electrically conductive TaO_*x*_ (*i.e*., σ_TaOx_ < 100 S m^–1^), Δ*V*_int_ becomes clearly lower as the Ta layer thickness increases (Figure 2(e)). For δ_Ta_ = 50 nm, if σ_TaOx_ is lower than 5 S m^–1^, Δ*V*_int_ eventually drops below the threshold voltage (region highlighted in cyan), meaning that no magneto-ionic effects will be induced in this case (while, for thinner Ta layers, for the same σ_TaOx_ value, Δ*V*_*i*_ will still be above the threshold). Note that during magneto-ionic motion of O^2–^ from Co_3_O_4_ to TaO_*x*_, the values of σ_TaOx_ will rapidly decrease, thus reducing the interface voltage and slowing down the magneto-ionic process. Another interesting effect is that when Co_3_O_4_ transforms to metallic Co, the conductivity of the magneto-ionic layer (σ_Co3O4_) increases. This, in turn, has an effect on Δ*V*_int_, which is shown in Figure 2(f). In bulk metallic Co, σ_Co_ can reach values >10^7^ S m^–1^.^52^ As plotted in Figure 2(f), when σ_Co3O4_ transforms to Co and σ_Co3O4_ increases, the interface voltage drastically drops, and Δ*V*_imt_ easily falls below the threshold voltage. Then any magneto-ionic response will tend to stop. As an example, for the particular case of δ_Ta_ = 50 nm, any eventual O^2–^ ion motion triggered while σ_TaOx_ is sufficiently large will tend to stop as soon as σ_Co3O4_ increases above 10^3^ S m^–1^ (see Figure 2(f)) or σ_TaOx_ decreases below 5 S m^–1^ (Figure 2(e)). It is noteworthy that, although this intuitive picture is a simplified representation of the reality, it already provides some basic understanding of the role of the TaO_*x*_ layer thickness and resistivity on the induced magneto-ionic effects.

**Figure 4:**
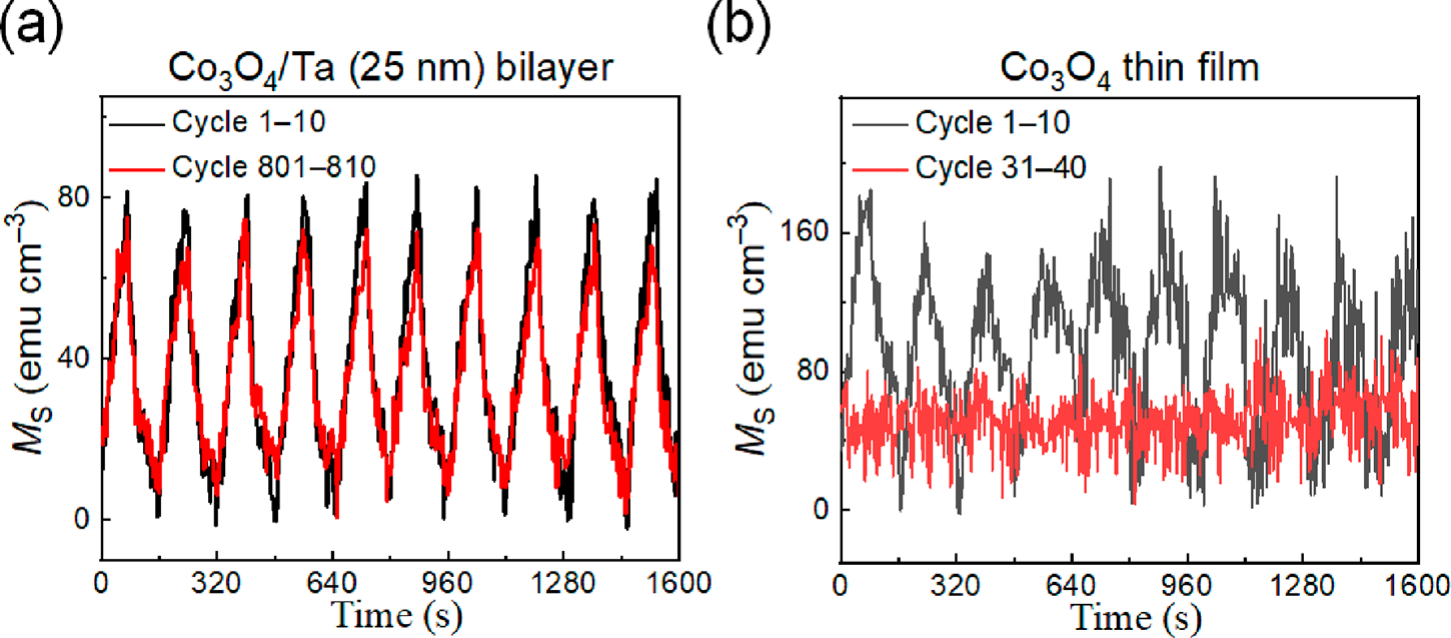
Magneto-ionic cyclability of (a) Co_3_O_4_/Ta (25 nm) bilayer films and (b) the Co_3_O_4_ thin films subjected to −25 V/+25 V voltage pulses applied with a periodicity of 80 s.

Notwithstanding the decrease of the steady-state *M*_S_ with the deposition of a Ta capping layer (by approximately 25% for δ_Ta_ = 25 nm), the formation of TaO_*x*_ (which acts as a solid electrolyte) has one very beneficial effect: it drastically enhances magneto-ionic cyclability. This is evidenced in Figure 4, which shows the cyclability results (*i.e*., repeated increase/decrease of *M*) in a Co_3_O_4_ thin film and Co_3_O_4_/Ta (25 nm) bilayer films upon application of −25 V/+25 V voltage pulses with a duration of 80 s. As shown in Figure 4(a) and (b), a very stable and reversible behavior is observed during the first 10 cycles for both samples. However, the Co_3_O_4_/Ta (25 nm) bilayer sample maintains a very stable cyclability even after 800 cycles (Figure 4(a)), whereas no sign of magneto-ionic effect is detected for Co_3_O_4_ films after 30 cycles (Figure 4(b)). This demonstrates a significant enhancement of the endurance of the system by the deposition of a Ta overlayer with appropriate thickness.

The improved cyclability in the Co_3_O_4_/Ta (25 nm) bilayer is ascribed to the role of Ta in allowing diffusion of oxygen ions. In this sample, when negative voltage is applied, the O^2–^ ions exiting Co_3_O_4_ are captured by Ta, forming a TaO_*x*_ solid electrolyte, instead of being directly released to the liquid electrolyte. Ta is a well-known oxygen getter,^53^ and TaO_*x*_ is a good ionic conductor.^54^ In unprotected Co_3_O_4_ films, O^2–^ ions are directly dissolved in the propylene carbonate, and once solvated with the PC chains, they can travel long distances toward the positively charged counter electrode (*i.e*., the Pt wire), where they eventually form O_2_ gas bubbles and are released to the atmosphere at sufficiently high voltages. This long-distance transport of ions in the liquid is the main reason for the poor cyclability of uncapped Co_3_O_4_ films. However, when a Ta interlayer is grown adjacent to Co_3_O_4_, O^2–^ ions move back and forth along relatively short distances (for alternating applied voltages of opposite polarities), and the process is confined between Co_3_O_4_ and the TaO_*x*_ solid electrolyte, thus drastically improving cyclability.

**Figure 5:**
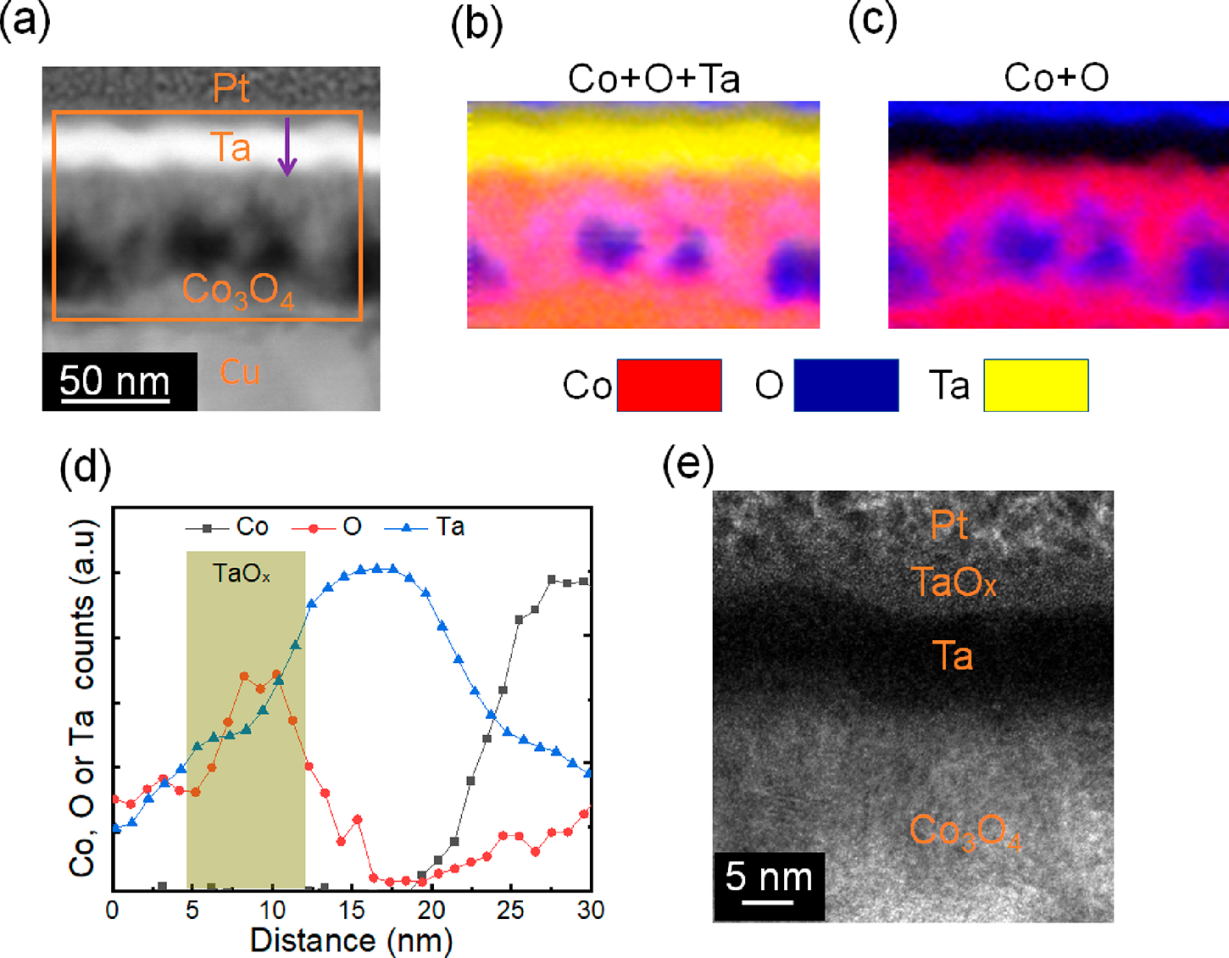
Compositional characterization of the Co_3_O_4_/Ta (25 nm) films gated at −25 V for 1 h. (a) HAADF-STEM and (b, c) elemental EELS mappings corresponding to the area marked with an orange rectangle in the HAADF-STEM image. Cu and Pt layers serve as working electrode and protective capping layer during TEM lamellae preparation, respectively. Co, O, and Ta are represented by red, blue, and yellow colors in the EELS elemental mappings. (d) Depth profile of Co, O, and Ta elements along the dark pink arrow drawn in (a). (e) High-resolution TEM image of the sample treated at −25 V for 1 h.

**Figure 6:**
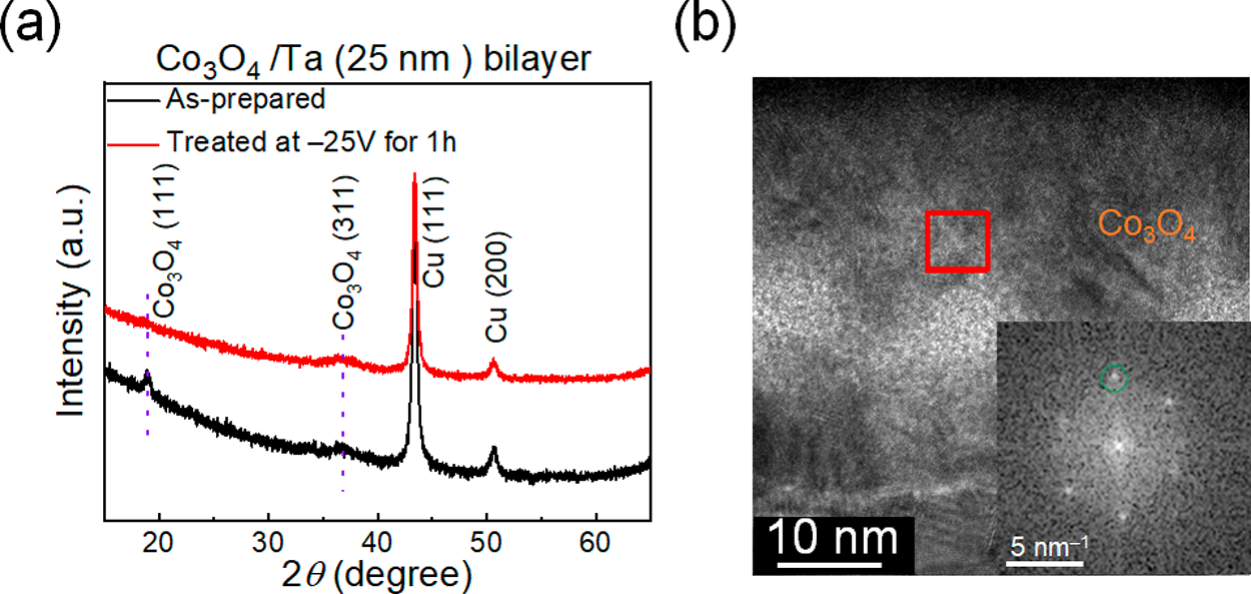
Structural characterization by X-ray diffraction (XRD) and high-resolution transmission electron microscopy (HRTEM). (a) θ/2θ XRD diffraction patterns of Co_3_O_4_/Ta (25 nm) films in the as-prepared state and after being treated at −25 V for 1 h. (b) HRTEM images of the cross section of the Co_3_O_4_/Ta (25 nm) film after applying −25 V for 1 h. The inset shows the fast Fourier transform (FFT) of the area marked with a red rectangle, and it shows spots with an average interplanar distance of 0.19 nm, which corresponds to (101) hexagonal-closed-packed (HCP) metallic Co. For phase identification, the cards no. PDF 00-005-0727 and PDF 00-009-0418 were taken for Co and Co_3_O_4_, respectively.

To further understand the effect of the top Ta layer on the induced magnetic properties from the perspective of the ion transport mechanism, cross-sectional lamellae of a bilayer sample (40-nm-thick Co_3_O_4_ plus 25-nm-thick Ta overlayer) electrolyte-gated at −25 V for 1 h were studied by HAADF-STEM and EELS. As shown in Figure 5(a)–(c), an inhomogeneous microstructure inside the Co_3_O_4_ layer is generated upon voltage application, with Co-rich and O-rich regions, leading to void-like morphologies, in agreement with previous studies on this kind of magneto-ionic material.^11^ In turn, an enrichment in O is observed in the upper part of the Ta layer. This is clearly evidenced by quantitative EELS analysis (Figure 5(d)) and high-resolution TEM (Figure 5(e)). This indicates that, besides the O^2–^ ion transport from Co_3_O_4_ toward and across the Ta layer, O^2–^ ions also locally migrate and redistribute inside the Co_3_O_4_ layer, eventually forming metallic Co.^11^ Interestingly, the mixing of O and Ta elements on the top part of the Ta layer demonstrates the oxidation of Ta. Considering that the liquid electrolyte used in this work provides a nonaqueous environment, the observed O signal can only originate from oxygen transport from Co_3_O_4_. The accumulation of O^2–^ on the top of the TaO_*x*_ layer suggests that ion transport remains rather restricted within the Co_3_O_4_/TaO_*x*_ bilayers (*i.e*., short-distance ion diffusion). In other words, the presence of the thin TaO_*x*_ solid electrolyte limits the amount of O^2–^ released to the liquid, thereby enhancing cyclability. Furthermore, during this magneto-ionic experiment, Co_3_O_4_ tends to become more amorphous. This is corroborated by θ/2θ X-ray diffraction (see Figure 6(a)), where the peak from Co_3_O_4_ (111) planes disappears upon voltage treatment. In addition, the FFT spots obtained from high-resolution TEM prove the existence of metallic Co in the treated sample, as can be seen in Figure 6(b). The formation of metallic Co is responsible for the *M*_S_ increase after voltage treatment (Figure 2(c) and Figure 3).

To assess the electrochemical behavior of the system, cyclic voltammetry (CV) curves were recorded for all the surfaces involved in the main magneto-ionic setup (see Figure S1 in the Supporting Information). When the Cu grown on Ti is exposed to the electrolyte, a cathodic wide peak is observed, centered at −0.9 V *vs* Pt reference electrode, while further anodic runs evidence a flat peak. This is consistent, in principle, with the reduction of Cu_2_O (native oxide layer formed on Cu when exposed to air) to Cu (first wave) and consequent oxidation of Cu to Cu_2_O. The CV curve of Co_3_O_4_ grown onto the Ti/Cu does not show the Cu waves, confirming that the cobalt oxide layer does cover completely the Cu phase and prevents its oxidation/reduction. Once Ta is grown on Co_3_O_4_, a tiny wide oxidation wave is observed, centered at 0.6 V *vs* Pt, in agreement with the expected oxidation of Ta. Since both coatings (Co_3_O_4_ and TaO_*x*_) are less conductive than Cu, the redox processes are hindered compared to the case of Cu.

**Figure 7:**
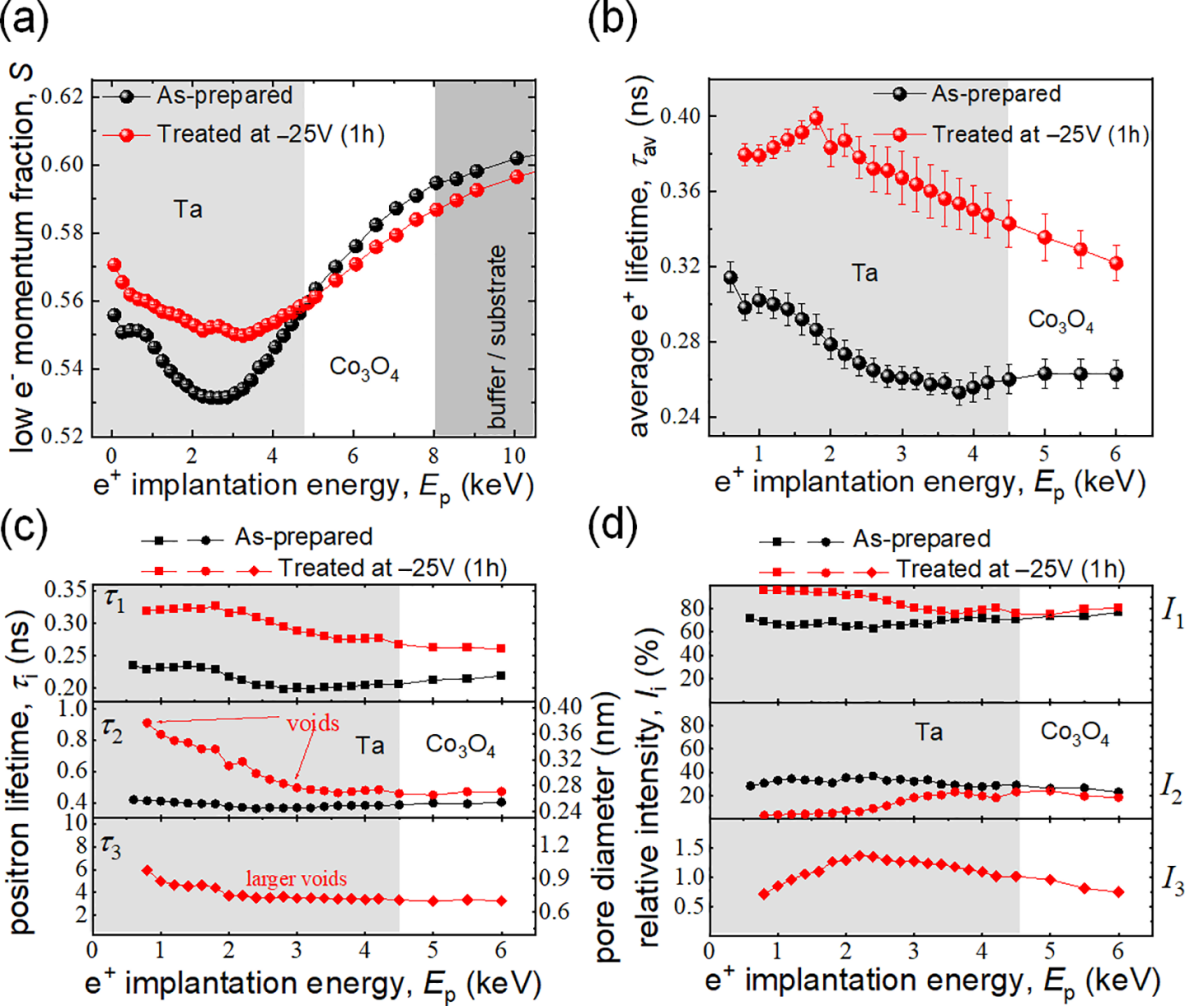
Positron annihilation spectroscopy characterization of the as-grown and voltage-treated (−25 V for 1 h) Co_3_O_4_/Ta (25 nm) sample. (a) Dependence of the low electron momentum fraction (*S*) on the positron implantation energy (*E*_p_). (b) Dependence of the average positron lifetime, τ_av_, on *E*_p_. (c) Dependence of the positron lifetime components (τ_i_) on *E*_p_. (d) Dependence of the relative defect type intensity (*I*_i_) on *E*_p._.

To shed further light on the microstructure of the films upon magneto-ionic actuation, Doppler broadening variable energy positron annihilation spectroscopy (DB-VEPAS)^55^ and variable energy positron annihilation lifetime spectroscopy (VEPALS)^56^ experiments were performed (see Experimental Section). PAS is a sensitive probe to open volume defects on atomic scales and across depth due to positrons’ preferential localization and subsequent annihilation with electrons in crystal empty spaces, *i.e*., vacancies and their agglomerates. The energy distribution and time necessary for annihilation depends on the local electron density.^57^ As shown in Figure 7(a), after negative biasing at −25 V, the low electron momentum fraction, *S* (directly proportional to the size and concentration of defects),^58^ increases in the Ta region but slightly drops in the Co_3_O_4_ layer, suggesting a substantial raise of defect density in Ta and a slight drop of defect concentration in Co_3_O_4_. The increase of defect density in Ta is probably a consequence of the O^2–^ interdiffusion and the formation of TaO_*x*_. Additionally, the average defect size (which is proportional to the average positron lifetime, τ_av_), strongly increases in both TaO_*x*_ and Co_3_O_4_ layers after voltage actuation (Figure 7(b)). The expected average size is in the range of large vacancy agglomerates (8–10 vacancies).^59^Figure 7(b) and (c) show the existence of three discrete lifetime components (τ_1,_ τ_2_, and τ_3_),^59^ which correspond to three different average defect sizes.^11^ They were obtained by deconvolution of PALS spectra using a nonlinear least-squared fitting method (package PALSfit software).^60^ The corresponding relative intensities (*I*_1_, *I*_2_, and *I*_3_) relate to the concentration of each defect type (size). The larger τ_*i*_ is, the larger the defect size is since it takes longer for positrons to be annihilated with electrons.^57^ In the as-grown sample, only τ_1_ and τ_2_ lifetime components are detected, meaning an absence of large void-like structures (no τ_3_) in the prebiasing state. The most abundant defect size (τ_1_) contributes as *I*_1_ = 62–77% of the overall signal and is in the range of bivacancy clusters in the upper subsurface Ta region (*i.e*., passivation layer) and single vacancy in the underneath Ta sublayer.^59^ τ_1_ ≈ 213 ps for Co_3_O_4_ represents a defect size involving 3–4 mixed (Co and O) vacancies within a complex, based on our previous works/publications.^11^ After biasing at −25 V, τ_1_ strongly increases both in Ta and Co_3_O_4_ layers. Open volume is generated in the size range of 4–6 vacancy agglomerations for Ta and >7–8 for Co_3_O_4_, which results in an increase of intensity to *I*_1_ = 75–95%. The second lifetime component, τ_2_, for the as-grown sample is in the range of large vacancy agglomerations (>10 vacancies), typical for grain boundaries, with corresponding *I*_2_ = 28–37% of positrons being annihilated at these defect states. Negative biasing increases τ_2_ to the range of voids (the threshold is usually about 500 ps) with an average diameter in the range of 0.28–0.37 nm,^61^ while their density is quite small, up to *I*_2_ ≈ 10%, and they do not reach deeper than the upper 10 nm of Ta. Clearly, these defects are associated with the formation of the upper TaO_*x*_ (Figure 5). Finally, a larger pore population (0.7–0.8 nm in diameter) was found after biasing (τ_3_), with low but not negligible intensity (*I*_3_ ≈ 1.4%), which extends into the Co_3_O_4_ layer. The existence of pore-related components (especially τ_3_) is a fingerprint of increasing amorphization of the TaO_*x*_ layer, whereas the Co_3_O_4_ layer remains less affected and nanocrystalline. The largest free volume is found in the direct vicinity of the surface, *i.e*., the most amorphized region. The increase of positron lifetimes in the Ta layer after biasing evidences the O^2–^ transport-generated expansion of available open volume channels, which enables large cyclability of the system.

## Conclusions

In summary, this work demonstrates the beneficial effect of adding a thin solid ionic conductor (in this case, a naturally passivated amorphous Ta interlayer) on the magneto-ionic response of Co_3_O_4_ films under the action of an electric field when immersed in liquid electrolytes. *A priori*, adding a capping layer between the Co_3_O_4_ film and the liquid electrolyte could be thought of as simply hindering magneto-ionics and reducing (or eventually suppressing) any oxygen ion migration triggered by the externally applied electrical voltage. This is indeed observed for thin metallic capping layers (*e.g*., 5 nm Pt) or thick highly resistive Ta layers (with a thickness larger than 50 nm). However, for thinner Ta interlayers, in spite of a moderate reduction of the steady-state *M*_S_ (by a factor of 25% for 25-nm-thick Ta), a drastic enhancement of magneto-ionic cyclability is observed, from less than 30 cycles in uncoated Co_3_O_4_ to more than 800 cycles for a Ta thickness of 25 nm. Such enhancement of endurance is ascribed to the key role of the generated TaO_*x*_ layer in preventing O^2–^ from being released to the liquid electrolyte, i.e., limiting oxygen ion transport within the Co_3_O_4_/TaO_*x*_ layers. This is confirmed by compositional/structural characterization using HAADF-STEM and EELS as well as positron spectroscopy experiments. Beyond magneto-ionics, the reported strategy to enhance cyclability can be easily extrapolated to other systems relying on ion transport mechanisms, such as iontronics, sensors, or neuromorphic computing.

## Experimental Section

### Sample Fabrication

Co_3_O_4_ thin films of 40 nm thickness were grown at room temperature by reactive sputtering in a high-vacuum chamber (with a base pressure of <8 × 10^–8^ Torr) on nondoped ⟨100⟩-oriented Si wafers previously coated with a 20-nm-thick titanium adhesion layer and 60-nm-thick copper seed layer. Prior to growing Co_3_O_4_, the Cu seed layers were partly masked to leave enough space for the electric contact (*i*.*e*., to later serve as a working electrode). The Co_3_O_4_ films were subsequently coated with Ta protective layers of variable thickness, ranging from 5 to 50 nm, which were left unprotected to self-passivate in air. Two reference films were also prepared to serve as references: uncoated Co_3_O_4_, and Co_3_O_4_ coated with Pt with the same range of thicknesses as Ta. The growth of Co_3_O_4_ was carried out in a mixed Ar and O_2_ atmosphere using an oxygen partial pressure of 50% and a total working pressure of 3 × 10^–3^ Torr. The distance between the substrate and targets was around 10 cm, and the growth rate was approximately 0.6 Å s^–1^. Ti, Cu, Ta, and Pt layers were grown under 3 × 10^–3^ Torr Ar. The gun power to grow Ta was 100 W.

### Magneto-Ionic Characterization and Cyclic Voltammetry Curves

Room-temperature magnetoelectric measurements were performed using a commercial vibrating sample magnetometer from Micro Sense (LOT, Quantum Design), with a maximum applied in-plane magnetic field of 2 T. The samples were electrolyte-gated using an external Agilent B2902A power supply, applying voltage between the counter electrode (a Pt wire) and the working electrode (*e.g*., the investigated Si/Ti/Cu/Co_3_O_4_/Ta thin films) in a homemade electrolytic cell (see Figure 2(b)). The electrolyte consisted of anhydrous propylene carbonate with Na^+^ and OH^–^ solvated species (10–25 ppm), formed by immersing small pieces of metallic sodium that were able to react with any possible traces of water.^11^ Negative voltages in this work indicate the accumulation of negative charges at the working electrode (and *vice versa* for positive voltages). The magnetization (*M*) was obtained by normalizing the magnetic moment to the sample volume exposed to the electrolyte. Note that the linear slopes in the hysteresis loops at high fields (arising from diamagnetic or paramagnetic contributions) were subtracted by correcting the background signal (*i.e*., at fields always significantly larger than the saturation fields).

Cyclic voltammetry curves for all the surfaces involved in the main magneto-ionic setup were also recorded. For these experiments, a scan speed of 10 mV/s and a potential sweep range from 0 V to −2 V to +1.4 V and to 0 V *vs* Pt were selected. The exposed surface was 0.5 cm^2^, and the distance between the reference and working electrode was 4 mm, thus mimicking the conditions utilized during the magneto-ionic experiments. Here, however, we used three electrodes (including a reference electrode), whereas magneto-ionic experiments were performed in a two-electrode configuration. Thus, all voltages in the CV curves are given with respect to a reference electrode, in this case Pt (99.99% Goodfellow), known to act well in organic solvents. The global cell (two-electrode) potential has been measured during these CV experiments to be on the order of 4 to 6 V depending on the system. Further potential could not be applied during the CV curves due to the limitations of the utilized potentiostat (VSP Biologic). However, the curves obtained in this potential range are already representative of the electrochemical behavior of the system.

### Structural and Compositional Measurements

θ/2θ XRD patterns were collected on a Materials Research diffractometer from Malvern PANalytical Company, equipped with a PIXcel^1D^ detector, using Cu Kα radiation. HRTEM, HAADF-STEM, and EELS were carried out on a TECNAI F20 HRTEM/STEM microscope operated at 200 kV. Cross-sectional lamellae were prepared by focused ion beam, placed onto a copper transmission electron microscopy grid, and topped with a protective platinum layer.

### Doppler Broadening Variable Energy Positron Annihilation Spectroscopy and Variable Energy Positron Annihilation Lifetime Spectroscopy

DB-VEPAS measurements were conducted at the setup for *insitu* defect analysis (AIDA)^55^ of the slow positron beamline (SPONSOR).^62^ Positrons were accelerated and mono-energetically implanted into samples in the range of *E*_p_ = 0.05–35 keV, which allows for depth-sensitive analysis. The mean positron implantation depth was approximated using a simple material density (ρ)-dependent formula: ⟨*z*⟩ = 36/ρ·*E*_p_^1.62^.^63^ Since at the annihilation site thermalized positrons have very small momentum compared to the electrons, a broadening of the 511 keV line is observed mostly due to momentum of the electrons, which is measured with a high-purity Ge detector (overall energy resolution of 1.09 ± 0.01 at 511 keV). This broadening is characterized by a parameter *S* defined as a fraction of the annihilation distribution in the middle (511 ± 0.93 keV). The *S*-parameter is a fraction of positrons annihilating with low-momentum valence electrons and represents vacancy-type defects and their concentration.^58^ VEPALS measurements were conducted at the monoenergetic positron source (MePS) beamline, which is an end station of the radiation source ELBE (Electron Linac for beams with high Brilliance and low Emittance) at Helmholtz-Zentrum Dresden-Rossendorf (Germany).^56^ A digital lifetime CeBr_3_ scintillator detector was used, with a homemade software employing an SPDevices ADQ14DC-2X with 14-bit vertical resolution and 2 GS s^–1^ (gigasamples per second) horizontal resolution and with a time resolution function down to about 0.230 ns.^64^ The resolution function required for spectrum analysis uses two Gaussian functions with distinct intensities depending on the positron implantation energy, *E*_p_, and appropriate relative shifts. All spectra contained at least 1 × 10^7^ counts. The spectra were deconvoluted using the PALSfit fitting software into discrete lifetime components, which directly confirm different defect types (*i*.*e*., sizes).^60^ Typical lifetime spectrum *N*(*t*) is described by *N*(*t*) = ∑(1/τ_i_)*I*_*i*_ exp(−*t*/τ_*i*_), where τ_*i*_ and *I*_*i*_ are the positron lifetime and intensity of the *i*th component, respectively (∑*I*_*i*_ = 1).

The corresponding relative intensities (*I*_*i*_) reflect to a large extent the concentration of each defect type, and positron lifetimes (τ_*i*_) are directly proportional to defect size (*i*.*e*., the larger the open volume, the lower the probability and the longer it takes for positrons to be annihilated with electrons). The positron lifetime and its intensity were probed as a function of positron implantation energy *E*_p_ or, in other words, implantation depth (thickness). The average positron lifetime τ_av_ is defined as τ_av_ = ∑_*i*_τ_*i*_·*I*_*i*_, which is the weighted average of the defect size. The shortest lifetime component (τ_1_ < 0.32 ns) represents positron annihilation inside vacancy clusters (likely within grains) and/or at the grain boundaries, depending on the film’s microstructure. The intermediate lifetime (0.35 < τ_2_ < 0.90 ns) accounts for annihilation at larger vacancy clusters (linked to grain boundaries and their intersections), surface states, and small voids/pores (0.28–0.37 nm in diameter, calculated based on the shape-free model for pore-size estimation of Wada *etal*.;^59^ the longest lifetime component (2.3 < τ_3_ < 3.3 ns) indicates contributions of larger voids (0.58–0.74 nm in diameter).

## Supporting Information Available

Cyclic voltammetry curves of the basic investigated layer structures (PDF)
