# Supplementary material for: Regulating Oxygen Ion Transport at the Nanoscale to Enable Highly Cyclable Magneto-Ionic Control of Magnetism
Source: ACS Nano. 2023 Mar 27;17(7):6973–84. doi: 10.1021/acsnano.3c01105 (PMC10100572; doi:10.1021/acsnano.3c01105)
Supplement: Supplementary file 1 — nn3c01105_si_001.pdf [file nn3c01105_si_001.pdf]

Supporting Information

Regulating oxygen ion transport at the nanoscale to enable highly cyclable magneto-ionic control of magnetism

Zhengwei Tan,[†] *Zheng Ma,*[†] *Laura Fuentes,*[‡,#] *Maciej Oskar Liedke,*[¥] *Maik Butterling,*[¥] *Ahmed G. Attallah,*[¥] *Eric Hirschmann,*[¥] *Andreas Wagner,*[¥] *Llibertat Abad,*[‡,#] *Nieves Casañ-Pastor,*[‡] *Aitor F. Lopeandia,*[†] *Enric Menéndez,**[*,†], *Jordi Sort**[†,□]

[†]Departament de Física, Universitat Autònoma de Barcelona, 08193 Cerdanyola del Vallès, Spain

[‡]Institut de Ciència de Materials de Barcelona, CSIC, Campus UAB, 08193 Bellaterra, Barcelona, Spain

[#]Centre Nacional de Microelectrònica, Institut de Microelectrònica de Barcelona-CSIC, Campus UAB, 08193 Bellaterra, Barcelona, Spain

[¥]Institute of Radiation Physics, Helmholtz-Zentrum Dresden - Rossendorf, Dresden 01328, Germany

Institució Catalana de Recerca i Estudis Avançats (ICREA), Pg. Lluís Companys 23, E-08010

Barcelona, Spain

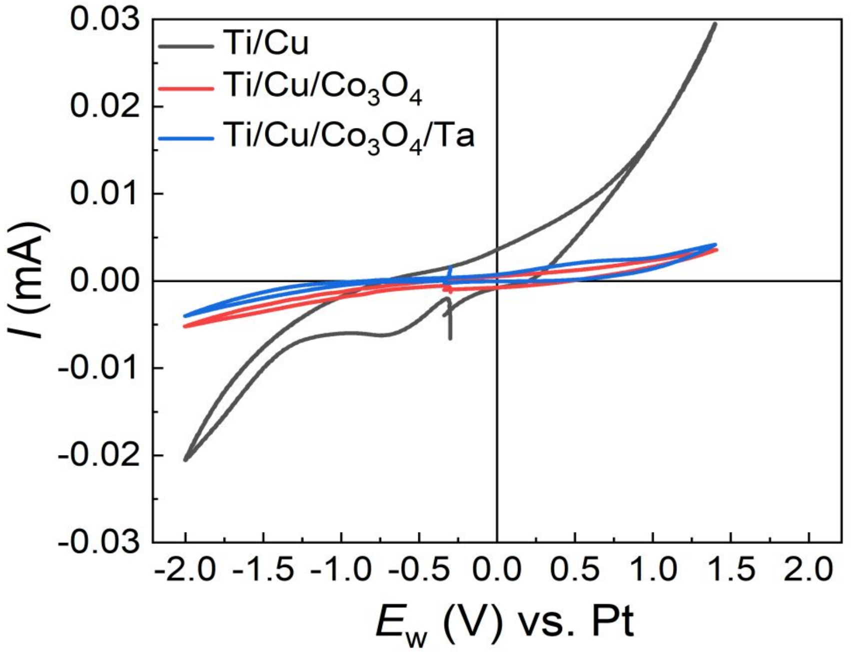

Figure S1: Cyclic voltammetry curves corresponding to the bare Ti/Cu conductive layers, the Ti/Cu/Co_3O_4 system and the Ti/Cu/Co_3O_4/Ta system.
